# ALWAYS TRY TO DO MY BEST: A THEMATIC ANALYSIS OF CHINESE AMERICAN DEMENTIA CAREGIVERS

**DOI:** 10.1093/geroni/igz038.2499

**Published:** 2019-11-08

**Authors:** Jinyu Liu, Bei Wu, Ada Mui, Yifan Lou, Wenxing Wei, Jiyun Jiang

**Affiliations:** 1 Columbia University, New York City, New York, United States; 2 New York University, New York, New York, United States; 3 Columbia University, New York, New York, United States

## Abstract

Objectives: Given the increasing prevalence of Alzheimer’s disease and related dementia (ADRD) in the United States and the rapid growth of the older Chinese American population, many older Chinese Americans are expected to need intensive care because of cognitive impairment. Prior studies on Chinese ADRD caregivers lack comprehensive examinations from a life course perspective that emphasizes the importance of time, context, process and meaning on human development. Using the life course perspective, this study aims to identify challenges and strength of caregiving experience for this population. Methods: We conducted semi-structured face-to-face interviews with 28 Chinese family caregivers of persons with ADRD in New York City. Thematic analysis method was used to assess the interview data. Results: Seven life-course themes emerged from the data. In the domain of challenges, four themes were identified: (1) physical and emotional exhaustion, (2) limited understanding on cognitive health, (3) difficulty in accessing effective and culturally-sensitive health care services for care recipients, and (4) caregivers’ inability to do self-care. Other three themes were found in the domain of strengths: (1) commitment to care due to cultural and religious values, (2) emotional closeness as resource to sustain caregiving, and (3) family support and cohesion. Conclusion: This study indicates that the life course perspective is an important lens to understand challenges and strengths of Chinese American caregivers. This study also suggests that health professionals could incorporate the life course perspective into assessment and intervention development when working with minority and immigrant ADRD family caregivers.

